# Can Heated Distilled Water Effectively Prevent Precipitate Formation between NaOCl and CHX?

**DOI:** 10.1155/2024/6612675

**Published:** 2024-01-06

**Authors:** Letícia Mendes Boppré, Julia Menezes Savaris, Emanuelle Catherine Maiola, Daniela Peressoni Vieira-Schuldt, Lucas da Fonseca Roberti Garcia, Cleonice da Silveira Teixeira, Eduardo Antunes Bortoluzzi

**Affiliations:** ^1^Department of Dentistry—Endodontics Division, Health Sciences Center, Federal University of Santa Catarina, Florianópolis, SC, Brazil; ^2^Department of Endodontics, College of Dental Medicine, Nova Southeastern University, Fort Lauderdale, FL, USA; ^3^Department of Diagnosis and Oral Health, Division of Endodontics, University of Louisville, Louisville, KY, USA

## Abstract

**Introduction:**

The present study aimed to investigate the capacity of different irrigation protocols using heated distilled water at 65°C (HDW), in preventing the formation of the brown–orange precipitate observed after the interaction between sodium hypochlorite (NaOCl) and chlorhexidine (CHX).

**Methods:**

Forty human canines were selected, prepared, and cleaved in two halves. Images of delimited areas in each root canal thirds were obtained through a stereomicroscope (16x and 40x). After reassembly, the teeth were distributed into four groups (*n* = 10) according to the final irrigation protocol: G1 (no HDW): EDTA + NaOCl + CHX with conventional irrigation (CI); G2 (HDW + CI): EDTA with passive ultrasonic irrigation (PUI) + NaOCl (PUI) + HDW (CI) + CHX (PUI); G3 (HDW + PUI): EDTA + NaOCl + HDW + CHX with PUI; G4 (HDW + CUI): EDTA (PUI) + NaOCl (PUI) + HDW with continuous ultrasonic irrigation (CUI) + CHX (PUI). After irrigation, the teeth were re-separated and images of the same delimited areas were obtained again. Scores were assigned according to the amount of precipitate observed, comparing the initial and final images. The data were submitted to Kruskal–Wallis, Dunn and Friedman statistical tests (*α* = 5%).

**Results:**

G1(no HDW) showed the highest scores in the analysis between groups (*p* < 0.001), with a greater amount of precipitate in the cervical and medium thirds (*p* < 0.001). The thirds of the other experimental groups did not differ from each other (*p* > 0.05).

**Conclusion:**

The intermediate irrigation with heated distilled water at 65°C prevented the formation of brown–orange precipitate, regardless of the use of ultrasonic activation (PUI or CUI).

## 1. Introduction

The use of irrigating solutions during endodontic procedures aims to promote debridement, cleaning, and disinfection of the root canal system [1]. No substance has all the ideal characteristics, therefore, the combined use of solutions in irrigation protocols has been suggested [1]. For example, the final irrigation using alternate applications of ethylenediaminetetraacetic acid (EDTA), a calcium-chelating agent, and sodium hypochlorite solution (NaOCl), an organic tissue solvent, was recommended after instrumentation to remove the smear layer from root canal walls [2]. Another protocol suggested the use of chlorhexidine gluconate (CHX) as the last irrigating solution to complement and maintain disinfection [3], due to its substantivity [4].

However, when more than one irrigant is used in sequence, they may still remain and come into contact with each other within the root canal system [5]. The chemical interaction between NaOCl and CHX may result in the brown–orange precipitate that causes staining of the dentinal structure [6]. It behaves like a chemical smear layer, which covers the dentinal tubules and interferes in the obturation sealing [7]. Also, the precipitate can contain para-chloroaniline (PCA), a potentially toxic substance [8].

Passive ultrasonic irrigation (PUI) and continuous ultrasonic irrigation (CUI) have also been recommended to improve the effects of irrigants on the root canal system [9, 10]. Both improves the dispersion of root canal irrigants via cavitation bubble implosions and/or acoustic streaming [11]. They have advantages over conventional irrigation (CI) with a syringe and needle, improving the penetration of solutions in isthmus and lateral canals [12].

Debridement of difficult-to-access canal areas may also be accomplished by reducing the surface tension and the viscosity of an irrigant by raising its temperature [13, 14]. The use of heated irrigants enhances their distribution in the root canal system and smear layer removal [9]. Dos Santos et al. [9] concluded that the use of heated distilled water at 65°C (HDW) as a final irrigant after the initial use of EDTA is as efficient in removing canal wall smear layer as NaOCl irrespective of whether CUI is used for simultaneous irrigant activation, with less deleterious effects on dentin microstructure. Knowing the cleansing power provided by HDW, it has been hypothesized that its use as an intermediate irrigant between the association of NaOCl and CHX it is beneficial to avoid the formation of the brown–orange precipitate.

Accordingly, this study aimed to evaluate the efficacy of the HDW as intermediate irrigation protocol between NaOCl and CHX, with and without ultrasonic activation (PUI or CUI), in preventing the formation of the brown–orange precipitate. The null hypothesis tested was that there would be no differences on the formation of the brown–orange precipitate after using HDW as intermediate irrigation protocol between NaOCl and CHX, regardless of the PUI or CUI use.

## 2. Materials and Methods

### 2.1. Sample Preparation

This research was approved by the local Human Research Ethics Committee (no. 3.413.317) and was conducted in full accordance with the ethical principals (World Medical Association Declaration of Helsinki, 2008). The sample size calculation was conducted (http://estatistica.bauru.usp.br/calculoamostral/ta_comparacao_multipla_independentes.php) considering a previous study with similar methodology [15]. The statistical power was set in 80%, alpha level of 5%, and the estimated standard deviation of 0.8. With a total of four groups, and based on the sample calculation results, 10 specimens were evaluated per group. Forty human canines with fully formed roots, a single straight root canal, and without previous endodontic treatment were selected. The reasons for the extraction of human teeth were unrelated to this research (such as periodontal disease). They were kept stored in distilled water until the experiments begin.

After access, the tooth length was obtained through the direct method by introducing a K#10 file (Dentsply Maillefer, Ballaigues, Switzerland) into the canal until its tip was seen in the apical foramen. The root of each tooth was surrounded by silicone (HydroXtreme, Swisstec, Coltene, Switzerland), in order to avoid the overflow of irrigating solutions. The working length (WL) was established 1-mm short from tooth length.

The root canal chemical–mechanical preparation was performed by a single operator, with a Reciproc R40 (40/.06) instrument (VDW, Munich, Germany), according to the manufacturer's instructions. The root canals were irrigated with 2.5% NaOCl (Rioquimica, SP, Brazil) through the CI, with a 5-mL syringe (Ultradent Products Inc., UT, USA) and calibrated needle 2-mm below the WL, with back-and-forth movements and simultaneous aspiration. Apical patency was maintained with a K#10 file taken to the apical foramen.

Then, grooves were made on the buccal and lingual faces of each tooth using double-sided diamond discs with 22-mm in diameter and 0.1-mm thick (ref. 7020, KG Sorensen), without penetrating the root canal. Jets of air cleared the debris. Each tooth was cleaved into two halves, mesial and distal, with the help of a hammer and chisel respecting the orientation of the grooves. In the middle of each third, a small circle was made, external to the canal, to be a reference point in obtaining the images. The samples were kept in an incubator at 37°C until they were taken for microscopic analysis.

### 2.2. Stereomicroscope Analysis

Two images were obtained with 16x and 40x magnifications from each root canal third, using a stereomicroscope (SteREO Discovery.V12, Carl Zeiss, Jena, Germany). These initial images were taken to verify the condition of the root canal walls, concerning their color, before the final irrigation protocols were carried out. The two tooth halves of each tooth were reassembled. The grooves previously created for cleavage were filled with a light-cured gingival barrier (Top Dam; FGM, SC, Brazil) to stabilize the parts. The reassembled tooth root was inserted into heavy condensation silicone impression material to increase stability and prevent leakage of the solutions used in the final irrigation protocols.

### 2.3. Final Irrigation Protocols

The 40 teeth were randomly assigned to four groups (*n* = 10), according to the different final irrigation protocols, that are summarized in Table 1.

*Group 1—control (no HDW):* The root canals were irrigated with 5 mL of 17% EDTA, for 60 s, (CI). Then, the irrigation proceeded with 5 mL of 2.5% NaOCl for 60 s (CI) and, finally, 5 mL of 2% CHX for 60 s (CI).

*Group 2 (HDW + CI):* The canals were irrigated with 2.5 mL of 17% EDTA for 30 s (CI), then activated by PUI for 30 s, and irrigated again with 2.5 mL of 17% EDTA for 30 s (CI). Then, the same irrigation protocol was performed with 2.5% NaOCl. After, were irrigated with 5 mL of HDW at 65°C for 60 s (CI) and, finally, with 2.5 mL of 2% CHX for 30 s + PUI for 30 s + 2.5 mL 2% CHX for 30 s (CI).

*Group 3 (HDW + PUI):* The irrigation technique used was the same as described in Group 2, except irrigation with HDW at 65°C, in which 2.5 mL was poured for 30 s (CI) + PUI for 30 s + 2.5 mL of HDW at 65°C for 30 s (CI).

*Group 4 (HDW + CUI):* The irrigation technique used was the same as described in Group 2, except irrigation with HDW at 65°C, which was poured into the canal using the CUI technique for 60 s.

During and at the end of each solution irrigation, the canals of all groups were aspirated through a metallic cannula positioned in the coronary access. PUI and CUI were performed using a specific insert (Irrisonic E1, Helse, SP, Brazil) positioned 1-mm below the WL, activated by ultrasound (JetSonic, Gnatus, SP, Brazil) at the 20% power indicated by the manufacturer, avoiding contact with the root canal walls.

The distilled water was heated on a hot plate (Thelga, Minas Gerais, Brazil). The water temperature remained constant (95°C) and monitored at all times using a thermometer immersed in the solution. The CUI technique followed the methodology described by Dos Santos et al. [9]. The HDW was poured into the root canal through a peristaltic pump. Through the path taken to the insert, the water lost temperature, reaching 65°C. The rate flow (3 mL/30 s) and the temperature of the poured water were verified and standardized according to a pilot study. For CI and PUI, water was deposited inside the root canal at 65°C.

### 2.4. Stereomicroscope and Scanning Electron Microscope (SEM) Analysis

**Figure 1:**
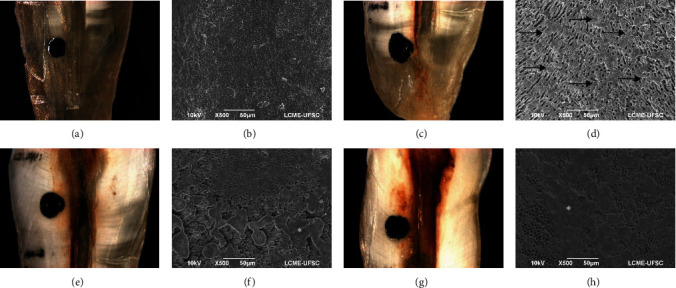
Representative images of scores under stereomicroscope in ×16 magnification (a, c, e, g) and scanning electron microscope in ×500 magnification (b, d, f, h). (a, b) Score 0: root canal third free of precipitate and with wide-open dentinal tubules. (c, d) Score 1: precipitate in less than half of the root canal third—arrows indicate the presence of the precipitate on the root dentin. (e, f) Score 2: precipitate covering more than half of the root canal third. (g, h) Score 3: root canal third completely covered by the precipitate.  ^*∗*^: indicates the presence of the precipitate.

After drying the root canals, new images of the same areas already photographed were obtained. The final images taken after irrigation were analyzed blindly by two previously calibrated examiners at two different moments with an interval of 1 week. The initial and final images were organized side by side for comparison purposes and the amount of brown–orange precipitate from each third was classified by scores [16]: 0—root canal third without precipitate; 1—precipitate present in less than half of the root canal third; 2—precipitate covering more than half of the root canal third; 3—root canal third completely covered by the precipitate (Figure 1). One specimen correspondent of each score was qualitatively evaluated under SEM, for illustration purpose, at ×500 magnification (Figure 1). The selected specimens were dried and sputter coated with a gold layer of 300 A° (Bal-Tec SCD 005, Bal-Tec Co., USA). The analysis was performed with the SEM (JEOL JSM 6390 LV, Akishima, Japan) set at 10.0 kV.

### 2.5. Statistical Analysis

The Kappa test was used to analyze the intra- and inter-examiner agreement. Data normality was checked with Shapiro–Wilk test. As the data shown nonnormality, for comparison between groups, the Kruskal–Wallis test was used. Once a statistical difference was detected, Dunn multiple comparison test was used to indicate between which groups these differences were. For the intragroup comparison, the Friedman test was used. Statistical analysis was performed using Jamovi software 1.6 (public domain) and BioEstat 5.0 (Mamirau Foundation, PA, Brazil). The level of significance adopted was 5%.

## 3. Results

The Kappa test indicated excellent intra and inter-examiner agreement, with values above 0.86 and 0.90, respectively.

The average rank, the median of the scores, and the results obtained from the comparison of the root canal thirds in the four groups are summarized in Table 2. In general, the G1 (no HDW) had significantly higher scores for precipitate formation (*p* < 0.05). The other experimental groups (HDW + CI, HDW + PUI and HDW + CUI) showed no differences between them. In the intragroup analysis, G1 (no HDW) had higher scores in the cervical and middle thirds when compared to the apical third (*p* < 0.05). No significant differences were observed between the root thirds in the other experimental groups (Table 2).

The qualitative analysis in SEM of the selected specimens showed correspondence with the patterns of precipitate formation, previously observed under a stereomicroscope (Figure 1). The specimens irrigated with HCW exhibited a cleaner dentin surface free of precipitates compared to G1 (no HDW).

## 4. Discussion

The brown–orange precipitate formation is due to the acid–base reaction between NaOCl and CHX [6]. For a safe and effective irrigation protocol using both solutions, it is imperative that the precipitate is avoided [17, 18]. Its formation implies dentinal tubules obliteration since it is deposited over the root canal walls, which can compromise the diffusion of intracanal medication and the obturation sealing [7], and stains root dentin [19]. Besides, the possibility of this precipitate diffusing into the periapical tissues should be considered since it may contain irritating and toxic components to the periapical tissues [8, 20].

Therefore, to avoid the formation of precipitate resulting from the interaction between NaOCl and CHX, the present study evaluated the effect of HDW to 65°C used as a neutralizing intermediate solution, with and without ultrasonic activation. The stereomicroscope results showed that the use of HDW, regardless of the applied irrigation technique, was able to prevent the precipitate formation.

In order to show that the root dentin staining did occur through the precipitate, a longitudinal evaluation of the specimens was performed, since the same area of interest was analyzed before and after the final irrigation protocols [9]. In addition, the analysis was made in stereomicroscope images which enabled the observers to see the staining clearly, without the need to perform any additional preparation on the samples surface [21].

The choice of a temperature of 65°C for heating the water was based on previous studies [9, 22, 23]. Furthermore, one of these studies by Sonntag et al. [22] observed that, after root canal irrigation, the preheated solution returns to body temperature (37°C) in an average time of 60 s, thus making it safe for use during endodontic treatment.

The distilled water was heated to reduce its surface tension and viscosity [13], to improve its reach, dispersion, and flow, especially in the anatomical complexities of the root canal system. Based on the results obtained, it is assumed that the heating has increased the water's capacity to remove the residual NaOCl deposited on root canal walls, so that the reaction with CHX is not observed. And together with the increase in temperature, the activation of irrigating solutions, through PUI and CUI, improves penetration and increases the flow of the solution, which consequently favors the contact of irrigating solutions with complex anatomical areas [10–12].

After the comparison between initial and final images, it was possible to confirm that the dentin staining occurred after the final irrigation. From the results, it was observed that in all thirds analyzed the specimens of the control group obtained the highest scores when compared to the experimental groups. Such result was expected, because no intermediate solution was used neither an activation method. There was no statistical difference between the other experimental groups, which shows that there is a similarity between the stirring techniques tested, when used together with heated distilled water.

A previous study showed that the use of different intermediate solutions failed to prevent the formation of byproducts [24]. Other studies also concluded that the distilled water, used as an intermediate irrigant with conventional irrigation, was inefficient in preventing the formation of the orangebrown precipitate on the root canal walls [25, 26]. One of those studies by Do Prado et al. [26] tested various protocols, including an intermediate flush with citric acid or phosphoric acid. After evaluation under SEM, the authors concluded that only the protocol using phosphoric acid as an intermediate irrigation completely prevented the formation of the chemical smear layer [26]. In none of these investigations [24, 26] did the authors use additional methods of agitating the solutions, which may have contributed to the results.

Considering the activation of the solutions, Keles et al. [21] observed successful results with different solutions, with and without activation, concerning the ability to remove the precipitate already formed from the surface of the root dentin. However, our goal was to avoid the formation of this byproduct, and not to remove it, ensuring greater predictability and safety to the procedure.

Regarding the thirds, the deposition of precipitate in the control group was concentrated in the cervical and middle thirds. It is assumed that the larger diameter of the dentinal tubules may have influenced these results, as it supposedly serves as a reservoir of irrigating solution remnants after aspiration. As there was no intermediate irrigation, this reservoir was not diluted by water, resulting in a greater amount of precipitate in these regions.

The results of the present study showed that HDW can be considered advantageous and useful, enabling the establishment of a final precipitate-free irrigation protocol, safely combining the studied irrigation solutions, enabling the use of chlorhexidine as the last irrigating solution.

Previous studies used the stereomicroscope to evaluate the presence of orange–brown precipitate [16, 21, 26] because it allows an accurate evaluation of color change in dentin walls, without covering or modifying the samples [26]. In this study, we also used the stereomicroscope for evaluation, however, it would be interesting to realize a further evaluation in SEMe in future studies, to evaluate the presence of chemical smear layer in a greater magnification, and the formation of precipitate inside the dentinal tubules [25].

## 5. Conclusion

The results of the current study indicate that distilled water at 65°C, when used as intermediate irrigation protocol between NaOCl and chlorhexidine solutions, was effective in preventing the formation of the brown–orange precipitate, regardless of the use of ultrasonic activation (PUI or CUI).

## Figures and Tables

**Table 1:** Groups distribution according to the final irrigation protocol.

Groups		17% EDTA			2.5% NaOCl			HDW			2% CHX		
	*n*	*v*	*t*	tUA	*v*	*t*	tUA	*v*	*t*	tUA	*v*	*T*	tUA
G1 (no HDW)	10	5	60	—	5	60	—	—	—	—	5	60	—
G2 (HDW + CI)	10	5	60	30	5	60	30	5	60	—	5	60	30
G3 (HDW + PUI)	10	5	60	30	5	60	30	5	60	30	5	60	30
G4 (HDW + CUI)	10	5	60	30	5	60	30	5	60	30	5	60	30

HDW, heated distilled water; CI, conventional irrigation; PUI, passive ultrasonic irrigation; CUI, continuous ultrasonic irrigation; *v*, solution volume (mL); *t*, application time (s); tUA, PUI and CUI application time (s).

**Table 2:** Mean rank scores, median of scores (in parentheses), and the results after statistical analysis using the Kruskal–Wallis test and the Dunn multiple comparison test by comparing root thirds in the four groups.

Groups	Root canal thirds		
	Cervical	Middle	Apical
G1 (no HDW)	56.5 (3.0)^Aa^	56.1 (3.0)^Aa^	55.2 (1.0)^Ab^
G2 (HDW + IC)	25.5 (0)^Ba^	23.5 (0)^Ba^	23.0 (0)^Ba^
G3 (HDW + PUI)	24.0 (0)^Ba^	22.0 (0)^Ba^	23.0 (0)^Ba^
G4 (HDW + CUI)	24.0 (0)^Ba^	28.2 (0)^Ba^	28.7 (0)^Ba^

HDW, heated distilled water; CI, conventional irrigation; PUI, passive ultrasonic irrigation; CUI, continuous ultrasonic irrigation. Lowercase superscript letters indicate a significant difference (Dunn test, *p* > 0.001) within a group among root thirds (row). Uppercase superscript letters indicate a significant difference (Dunn test, *p* > 0.001) among groups within a root third (column).

## Data Availability

The corresponding author can provide access to the datasets generated and/or analyzed during the current study upon a reasonable request.
